# The use of a clip prior to neoadjuvant chemotherapy for breast cancer with microcalcifications may not always be required

**DOI:** 10.1007/s10549-024-07517-1

**Published:** 2024-10-17

**Authors:** Henri Talec, Christophe Aubé, Catherine Guerin-Charbonnel, Pierre Berge

**Affiliations:** 1 Department of Radiology and Medical Imaging, University Hospital, 49100 Angers, France; 2 Institut de Cancérologie de l’Ouest, 44805 Saint-Herblain, France; 3 Institut de Cancérologie de l’Ouest, 49055 Angers, France

**Keywords:** Breast cancer, Microcalcification, Marker clip, Mammography

## Abstract

**Purpose:**

Neoadjuvant chemotherapy is now a common first line therapy for breast cancer. International guidelines recommend placement of a clip before commencement of therapy to assist with localizing the tumor bed in the event of excellent response—this takes up time and resources. The microcalcifications associated usually persist after chemotherapy and could serve as an alternative marker. We investigated to determine prognostic criteria to avoid the need for a marker clip before neoadjuvant chemotherapy for breast tumors associated with microcalcifications.

**Methods:**

We performed a 7 year single-center bi-site retrospective analytical observational study of 88 women with calcified breast carcinoma treated by neoadjuvant chemotherapy at our bi-site institution between September 2015 and September 2022. This study includied two groups (clip-free tumor localization vs. clip-free tumor non-localization), and investigating quantitative and qualitative predictive factors. The clip-free tumor localization after neoadjuvant chemotherapy was defined by the visibility of residual calcifications on both views of the pre-operative mammogram on the day of or the day prior to surgery.

**Results:**

The mean age of the 88 women included in our population was 52.8 years (± 12.7 years standard deviation). Of the 90 tumors with microcalcifications, 64 carcinomas (71.1%) were localizable with no marker clip after neoadjuvant chemotherapy. The main predictive factors significantly associated with clip-free tumor localization were number of calcifications > 10 (*P* < 0.0001), grade 2 tumor (*P* = 0.003) with a probability of locating tumor after neoadjuvant chemotherapy of 97.9%, 95% CI [95.6; 99.0].

**Conclusion:**

More than 10 microcalcifications in a grade 2 breast tumor at the initial diagnosis may obviate the need for a marker clip.

## Introduction

Neoadjuvant chemotherapy is now a common first line therapy for high risk breast cancers that progress rapidly or are inaccessible to immediate conservative treatment, and can result in better surgical options with increased breast conservation (conservative treatment rather than radical mastectomy) [1–3].

When neoadjuvant chemotherapy is initiated for a breast tumor, the carcinomatous tissue component may undergo partial or complete regression (some tumors do not respond or may indeed progress). International guidelines recommend placement of a marker clip inside the tumor before commencement of chemotherapy to facilitate pre-operative radiologic wire localization in the event of excellent response. This method is considered reliable and safe [3, 4], but has certain disadvantages such as possible complications, medical time consuming and a relatively high material cost of $85 to $320 per clip [5, 6].

**Fig. 1:**
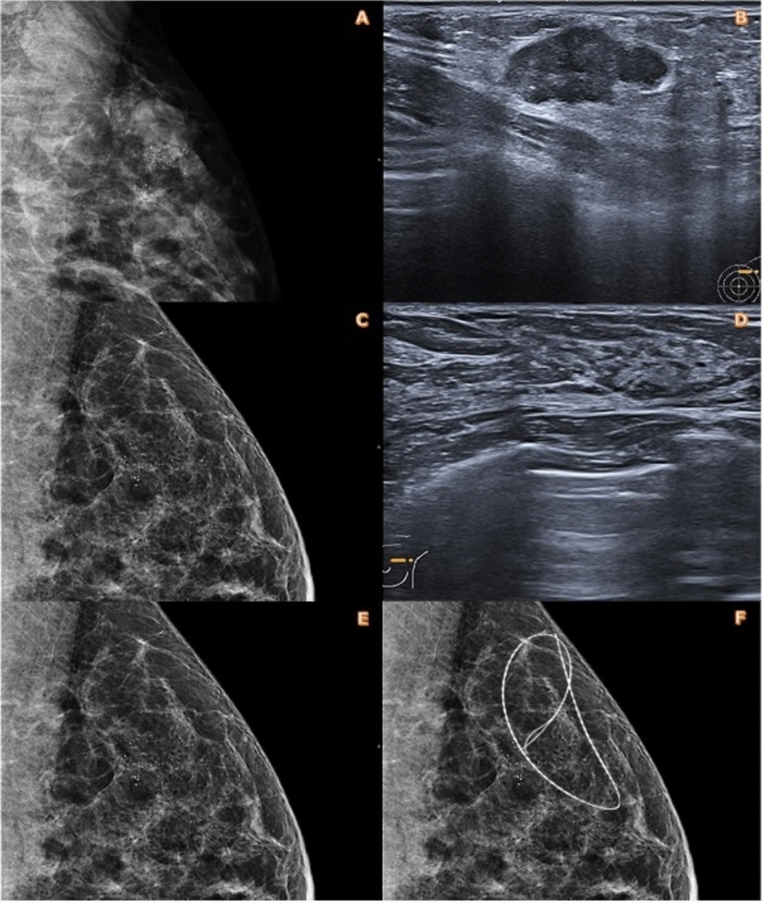
Invasive carcinome of no special type (ICNST) grade 2 with groups of microcalcifications > 10 units on mammography and ultrasound before (**A**, **B**) and after (**C**, **D**) neoadjuvant chemotherapy. Note the complete regression of the carcinomatous tissue component and the persistence of the micro-calcifications making it possible to place pre-operative radiologic wire localization after therapy (**E**, **F**)

When microcalcifications are associated with the tumor, they usually show no morphologic change after chemotherapy. They may increase or decrease in number, more rarely disappear [7–16], and so they could be used as a radiographic marker [4, 7], which could then be substituted for the marker clip (Fig. 1).

At present, no criteria have been formally defined for using or not using a marker clip prior to neoadjuvant chemotherapy for tumors with microcalcifications.

This raises the question of the systematic need for a marker clip, and the definition of objective criteria for determining whether it is necessary.

To the best of our knowledge, no studies specifically addressing this issue have been reported in the literature.

The main objective of our study was to determine the prognostic criteria for calcifications, tumors, or patients, to avoid the need for a marker clip. The secondary objective was to evaluate the inter-observer reproducibility of these criteria.

## Methods

Access to health information was approved by an ethics committee (#2022-158, Ethic commitee CHU d’Angers). All patients had approved the re-use of their electronically recorded data. Guidelines for reporting this study were derived from the “Strengthening the Reporting of Observational Studies in Epidemiology” (STROBE) statement.

### Study design and population

We performed a retrospective single-center bi-site analytic observational cohort study of consecutive patients with a breast carcinoma treated by neoadjuvant chemotherapy admitted during 84 month period (September 1, 2015 to September 30, 2022) at our bi site oncology institution. Inclusion criteria were women older than 18 years, breast carcinoma confirmed by diagnostic micro or macrobiopsy, and treated by neoadjuvant chemotherapy. Exclusion criteria were the absence of one or more suspicious microcalcifications associated tumor, missing mammographic or histologic data. This study included two groups (clip-free tumor localization vs. clip-free tumor non-localization) with research into quantitative and qualitative predictive factors.

The primary endpoint was to assess the no need for a marker clip after neoadjuvant chemotherapy, determined on the console by the visibility of residual calcifications in both the un-magnified pre-operative mammograms taken on the day of or the day prior to surgery.

The secondary endpoint included evaluation of inter-observer agreement for statistically significant predictive factors and the primary endpoint.

### Data collection and outcome assessment

All tumors of our studies received a marker clip according to the international guidelines. For each patient, all data relating to potential predictive factors were collected prior to neoadjuvant chemotherapy. Mammographic features were collected by console reading on craniocaudal, mediolateral oblique and magnified diagnostic mammograms. The histologic results were collected on original histology reports from the diagnostic biopsy, simulating a real-life condition.

Patient data were age and breast density (BI-RADS 5th Edition: A, B, C, D). Calcification data were group size (millimeters); number of calcifications associated with the tumor; suspicious morphology (BI-RADS 5th Edition: amorphous, fine pleiomorphic, coarse heterogenous, linear or brancher, round or punctiform); distribution (BI-RADS 5th Edition: grouped, diffuse, linear, regional, segmental); contrast with the underlying breast density; spread or not beyond the mass. Tumor data were size (millimeters); histology: invasive carcinome of no special type (ICNST), invasive lobular carcinoma (ILC), other, with or without in-situ associated; grade; percentage of hormonal receptors; HER2 status: positive (defined as 3 + staining or 2 + FISH amplified) or negative; Ki67 proliferation index.

The BI-RADS 5th edition lexicon was used, and all features were double-read by both a radiology resident (H.T) and a senior radiologist (P.B.) specializing in senology (4 years’ experience). In the event of mismatch, a joint re-reading was carried out to reach a consensus, and statistic analyses were carried out based on the consensus. Agreement was measured a posteriori, based on the initial analyses by each reader, on mammographic variables that were statistically significant, and on the primary endpoint.

### Statistical analysis

For all tests, statistic significance level was set at 5%. Analyses were performed (C.G.) with R software [R Core Team (2014). R: A language and environment for statistical computing. R Foundation for Statistical Computing, Vienna, Austria. URL: http://www.R-project.org/.]

The approach focused on the tumor studied, and tumors from the same patient were considered independent in terms of the features observed.

Quantitative variables were described by median, inter-quartile range, minimum, maximum, mean and standard deviation, and qualitative variables by number and percentage.

The impact of the different variables on the need for a clip was estimated using univariable and multivariable logistic models.

Stepwise procedure based on the Akaike Information Criterion (AIC) was used to define an optimal multivariable model, all variables with a univariable *P* value < 0.15 were entered into the initial model.

An unweighted Cohen’s kappa was used to agreement measure of qualitative variables, and intra-class correlation for ordinal variables.

Following this last analysis, a multivariable model with only objective variables and a minimal multivariable model were built.

## Results

### Patient demographics

**Fig. 2:**
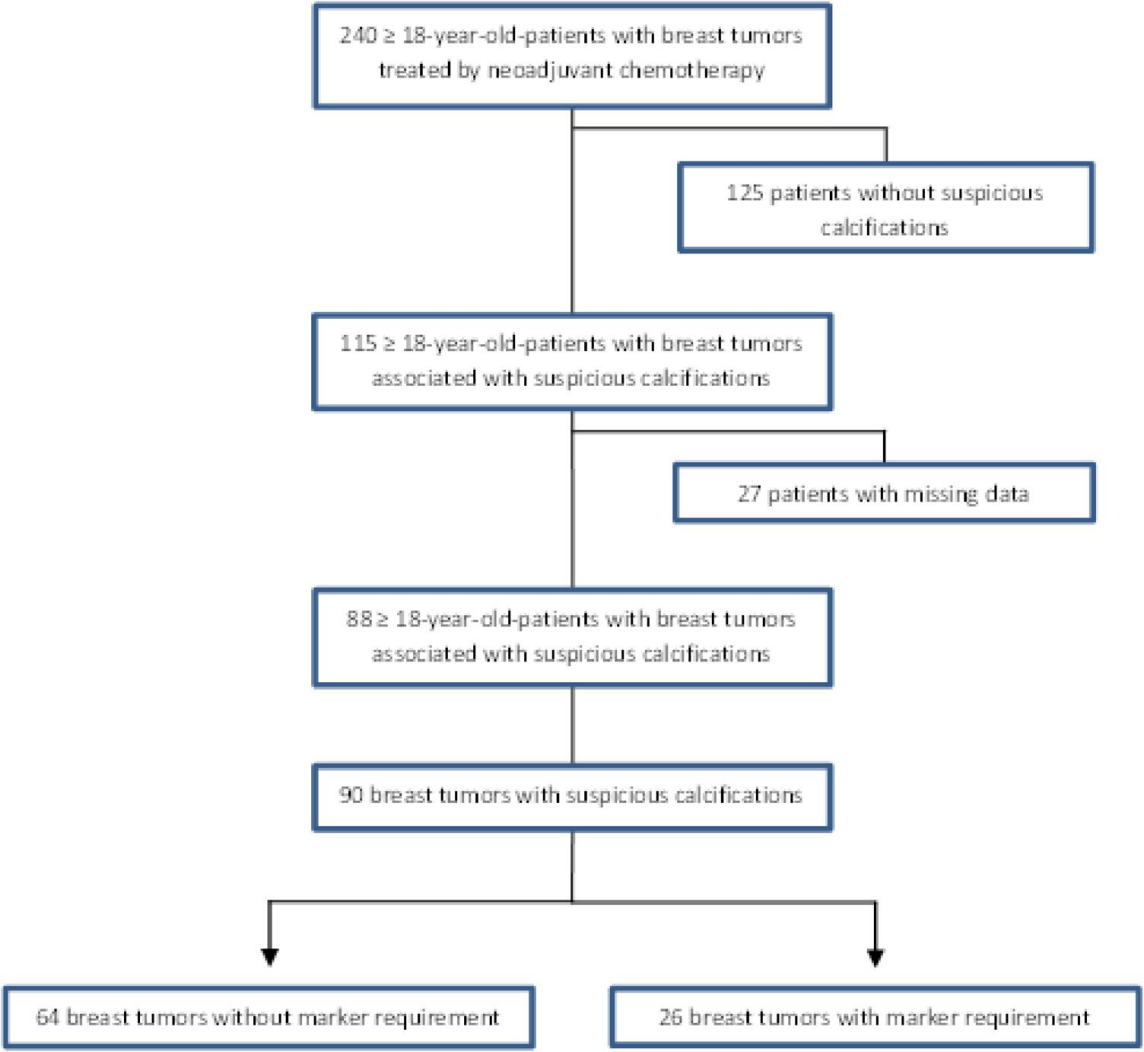
Flow-chart

After reviewing 240 medical records of adult women with breast carcinoma treated by neoadjuvant chemotherapy and excluding 125 patients with no suspicious calcifications associated (52% of patients), 25 patients with missing mammographic data (10% of patients) and 2 patients with missing histologic data (1% of patients), the population included 88 patients for a study sample of 90 tumors (Fig. 2*)*.

The mean age was 52.8 years (± 12.7 years). Type A breast density represented 9% of cases (8/90), type B 57% (51/90), type C 30% (27/90), and type D 4.5% (4/90): Table 1.

**Table 1:** Demographic table

Parameter	Value
No of participants	88
Age (years)*	52.84 ± 12.69
Breast density	
A	8 (8.89%)
B	51 (56.67%)
C	27 (30.00%)
D	4 (4.44%)
Mass size (mm)*	26.31 ± 13.06
Invasive or in-situ	
Invasive	51 (56.67%)
Both	39 (43.33%)
Histology	
ICNST	86 (95.56%)
ILC	3 (3.33%)
Other	1 (1.11%)
Grade	
1	0 (0.0%)
2	47 (52.22%)
3	43 (47.78%)
ER (%)	
< 10	34 (37.78%)
≥ 10%	56 (62.22%)
PR (%)	
< 10%	46 (51.11%)
≥ 10%	44 (48.89%)
HER2	
Negative	43 (47.78%)
Positive**	47 (52.22%)
Ki67 (%)*	30.63 ± 22.29
*nb. NA*	*9*
Group of Ca size (mm)*	25.71 ± 22.20
Ca number	
≤ 10	18 (20.00%)
> 10	72 (80.00%)
Morphology	
Amorphous	19 (21.11%)
Fine pleiomorphic	19 (21.11%)
Coarse heterogeneous	19 (21.11%)
Linear or branched	16 (17.78%)
Round or punctiform	17 (18.89%)
Distribution	
Grouped	40 (44.44%)
Diffuse	4 (4.44%)
Linear	7 (7.78%)
Regional	20 (22.22%)
Segmental	19 (21.11%)
Contrast Ca/breast	
Weak	34 (37.78%)
Strong	56 (62.22%)
Ca spread	
No	48 (53.33%)
Yes	42 (46.67%)

Unless otherwise specified, data are categoric variables presented as numbers, with percentages in parentheses

*Ca* calcifications, *ER* estrogen receptor, *HER2* human epidermal growth factor receptor-2, *ICNST* invasive carcinome of no special type, *ILC* invasive lobular carcinoma, *PR* progesterone receptor

*Data are means ± SDs, **3 + stained or 2 + FISH amplified

The mean size of the 90 tumors was 26.3 mm, with 57% invasive (51/90) and 43% invasive-in situ (39/90). Most tumors (96%) were ICNST (86/90), versus 3% ILC (3/90), and only 1 cancer (1%) with a different histologic type (metaplastic carcinoma). There were no grade 1 tumors in our population, grade 2 made up 52% (47/90) and grade 3 48% (43/90) of carcinomas. 62% of the tumors had ≥ 10% estrogen receptors (56/90), 49% had ≥ 10% progesterone receptors (44/90). HER2 positive was reported in 52% of cases (47/90) and the mean Ki67 value was 31% (SD: 22).

The mean size of calcification groups was 25.7 mm (SD: 22). Eighty percent of the groups had more than 10 calcifications (72/90). Amorphous, fine pleomorphic, and coarse heterogeneous morphologies predominated in equal proportions: 21% of groups of calcifications (19/90), fine linear or linear-branching morphology predominated in 18% of the groups (16/90), and round or punctiform morphology in 19% of the groups (17/90).

Diffuse distribution was found in 4% of cases (4/90), grouped distribution in 44% (40/90), linear distribution in 8% (7/90), regional distribution in 22% (20/90), and segmental distribution in 21% (19/90). Calcifications contrast with underlying breast density was considered strong in 62% of cases (56/90).

Groups of microcalcifications spread beyond the mass in 47% of cases (42/90).

The average delay between diagnostic mammography and pre-operative mammography was 184 days.

### Prognostic criteria for clipless localization

#### Univariable analysis

No patient feature (age; breast density) was statistically significant for clip-free tumor localization after neoadjuvant chemotherapy: Table 2*.*

**Table 2:** Univariable and multivariable results for available parameters; parameters included in stepwise multivariable model are in bold font

Parameter	Clip-free tumor non localization (*n* = 26)	Clip-free tumor localization (*n* = 64)	Univariable results		Multivariable results	
			Odds-ratio (IC95%)	*P* value	Odds-ratio (IC95%)	*P* value
Grade						
2	7 (26.92%)	40 (62.50%)				
3	19 (73.08%)	24 (37.50%)	0.22 [0.08; 0.58]	**0.0032**	0.01 [0.00; 0.13]	***0.0021***
Distribution						
Grouped	17 (65.38%)	23 (35.94%)			*Reference category: grouped or diffuse or linear	
Diffuse	4 (15.38%)	0 (0.00%)	–	–		
Linear	2 (7.69%)	5 (7.81%)	1.85 [0.35; 14.00]	0.4930		
Regional	2 (7.69%)	18 (28.12%)	6.65 [1.62; 45.48]	**0.0195**	28.15 [3.07; 526.22]	***0.0097***
Segmental	1 (3.85%)	18 (28.12%)	13.30 [2.38; 251.00]	**0.0162**	51.06 [3.27; 2114.49]	***0.0144***
Contrast Ca/breast						
Weak	14 (53.85%)	20 (31.25%)				
Strong	12 (46.15%)	44 (68.75%)	2.57 [1.01; 6.65]	**0.0481**	12.36 [1.99; 131.75]	***0.0146***
Number of Ca						
≤ 10	16 (61.54%)	2 (3.12%)				
> 10	10 (38.46%)	62 (96.88%)	49.60 [11.88; 346.55]	** < 0.0001**	835.61 [41.48; 79,035.23]	***0.0003***
Ca spread						
No	19 (73.08%)	29 (45.31%)				
Yes	7 (26.92%)	35 (54.69%)	3.28 [1.25; 9.39]	**0.0196**	0.11 [0.01; 0.69]	***0.0319***
Invasive/in situ						
Invasive	18 (69.23%)	33 (51.56%)				
Both	8 (30.77%)	31 (48.44%)	2.11 [0.82; 5.80]	**0.1290**		
ER (%)						
< 10	13 (50.00%)	21 (32.81%)				
≥ 10%	13 (50.00%)	43 (67.19%)	2.05 [0.81; 5.24]	**0.1306**		
Morphology						
Amorphous	8 (30.77%)	11 (17.19%)				
Fine pleiomorphic	6 (23.08%)	13 (20.31%)	1.58 [0.42; 6.17]	0.5023		
Coarse heterogeneous	2 (7.69%)	17 (26.56%)	6.18 [1.27; 46.35]	**0.0385**		
Linear or branched	1 (3.85%)	15 (23.44%)	10.91 [1.65; 218.14]	**0.0349**		
Round or punctiform	9 (34.62%)	8 (12.50%)	0.65 [0.17; 2.41]	0.5164		
Underlying breast density of Ca						
Strong	16 (61.54%)	20 (31.25%)				
Weak	10 (38.46%)	44 (68.75%)	3.52 [1.38; 9.37]	**0.0095**		
Age (years)*	51.62 ± 12.30	53.34 ± 12.91	1.01 [0.98; 1.05]	0.5566		
Breast density						
A	1 (3.85%)	7 (10.94%)				
B	13 (50.00%)	38 (59.38%)	0.42 [0.02; 2.68]	0.4340		
C	10 (38.46%)	17 (26.56%)	0.24 [0.01; 1.66]	0.2148		
D	2 (7.69%)	2 (3.12%)	0.14 [0.00; 2.24]	0.1837		
Mass size (mm)*	24.23 ± 14.78	27.16 ± 12.32	1.02 [0.98; 1.06]	0.3378		
PR (%)						
< 10	14 (53.85%)	32 (50.00%)				
≥ 10%	12 (46.15%)	32 (50.00%)	1.17 [0.47; 2.94]	0.7409		
HER2						
Negative	15 (57.69%)	28 (43.75%)				
Positive**	11 (42.31%)	36 (56.25%)	1.75 [0.70; 4.49]	0.2324		
Ki67 (%)*	33.17 ± 22.61	29.62 ± 22.27	0.99 [0.97; 1.01]	0.5161		
Group of Ca size (mm)*	23.35 ± 33.46	26.67 ± 15.77	1.01 [0.99; 1.03]	0.5196		

Unless otherwise specified, data are categoric variables presented as numbers, with percentages in parentheses

*Ca* calcifications, *ER* estrogen receptor, *HER2* human epidermal growth factor receptor-2, *PR* progesterone receptor

*Data are means ± SDs, **3 + stained or 2 + FISH amplified

Except grade, no tumor feature (size; histology; PR; ER; HER2; Ki67) was statistically significant for clipless localization after neoadjuvant chemotherapy. Only grade 3 (*reference*: grade 2) was not in favor of clipless tumor localization (*P* = 0.003, OR < 1)*.*

A group of microcalcifications composed strictly of more than 10 units was associated with clip-free tumor localization after neoadjuvant chemotherapy (*P* < 0.0001)*.*

Regional or segmental distribution was associated with clip-free tumor localization (*ref*: grouped distribution), with respectively *P* = 0.02 and *P* = 0.02. Strong calcifications contrast with the underlying mammary gland was associated with clip-free tumor localization (*P* = 0.048). Spread of calcifications beyond the mass was associated with tumor localization (*P* = 0.02). Coarse heterogeneous and fine linear or linear-branching morphologies were associated with clip-free tumor localization (*ref*: amorphous morphology), with respectively *P* = 0.04 and *P* = 0.04. No statistically significant differences were found for fine pleiomorphic or punctuate predominant morphologies. Group of microcalcifications size was not statistically significant for clip-free localization (Table 2).

#### Multivariable analysis

Grade 3 tumor (*P* = 0.02), regional distribution (*P* = 0.001) and segmental distribution (*ref*: grouped, diffuse or linear distribution) (*P* = 0.01), strong contrast of calcifications (*P* = 0.01), number of calcifications > 10 units (*P* < 0.001) and calcifications spread (*P* = 0.03) were associated with tumor localization in the stepwise multivariable model (Table 2).

### Inter-observer reproducibility

Agreement of clip-free tumor localization after neo-adjuvant chemotherapy was total with a coefficient of 1: Table 3.

**Table 3:** Inter-observer reproducibility

Parameter	Agreement coefficient
Clip-free tumor localization	1
Number Ca	
≤ 10 vs. > 10	0.81
Distribution	
Regional	0.41
Segmental	0.28
Contrast Ca/breast	0.61
Ca spread	0.87

*Ca* calcifications

After categorizing the number of calcifications in the group into]0;10] and > 10, agreement was estimated as strong with a coefficient of 0.81.

Agreement of regional or segmental calcification distribution was moderate and weak, with coefficients of 0.41 and 0.28, respectively.

The agreement of contrast factor was high at 0.61, while that for spread calcification factor was strong at 0.87.

### Multivariable model based on objective parameter (strong agreement)

Grade 3 tumors (*P* = 0.007) and a group of microcalcifications composed strictly of more than 10 units (*P* < 0.0001) were significantly associated with tumor localization after neoadjuvant chemotherapy in the stepwise multivariable model with objective parameters (histologic and mammographic parameters showing strong inter-observer agreement): Table 4.

**Table 4:** Multivariable model with objective parameters

Parameter	Odds-ratio (IC95%)	*P* value
Number of Ca		
> 10 vs. ≤ 10	204.37 [24.08; 5238.98]	< 0.0001
Grade		
3 vs. 2	0.05 [0.00; 0.30]	0.0065
Ca spread		
Yes vs. no	0.60 [0.13; 2.43]	0.4843

*Ca* calcifications

Retaining only significant parameters, a minimal multivariable model can be derived including only grade and number of microcalcifications > 10: Table 5.

**Table 5:** Minimal multivariable model

Parameter	Odds-ratio (IC95%)	*P* value
Number of Ca		
> 10 vs. ≤ 10	153.90 [21.52; 3530.88]	< 0.0001
Grade		
3 vs. 2	0.06 [0.00; 0.31]	0.0074

*Ca* calcifications

Based on this model, the probability of locating a grade 2 tumor with more than 10 calcifications after neoadjuvant chemotherapy is 97.9%, 95% CI [95.6; 99.0].

## Discussion

To the best of our knowledge, no similar studies specifically addressing criteria for not using a marker clip in breast tumor with calcifications have been reported in the literature. We identified various factors to favor clip-free tumor localization after neoadjuvant chemotherapy, such as a group of microcalcifications composed strictly of more than 10 units (*P* < 0.0001), grade 2 tumors (*P* = 0.003), regional (*P* = 0.02) or segmental (*P* = 0.02) distribution, strong contrast of calcifications on underlying breast density (*P* = 0.048), and spread of calcifications beyond the mass (*P* = 0.001).

The number of calcifications associated with the breast tumor at initial diagnosis is a fundamental predictive factor for clip-free localization. A group of microcalcifications composed strictly of more than 10 units is significantly associated with its localizability, with a probability of locating grade 2 tumors after neoadjuvant chemotherapy of 97.9%, 95% CI [95.6; 99.0].

This study suggests that a grade 3 tumor is a limiting factor for clip-free localization. We explain this by the aggressive and evolutive character of grade 3 tumors, resulting in an accelerated response to chemotherapy, making possible partial or complete disappearance of calcifications [17]. Not surprisingly, strong calcification contrast with the underlying breast density, because of their better visibility, is statistically significant in both univariable and multivariable analyses.

All objective variables in this study are reproducible with strong agreement like number of calcifications (coefficient of 0.81) and clip-free tumor localization (coefficient of 1). Indeed, all that was required for the primary endpoint was observation of one residual microcalcification after neoadjuvant chemotherapy to locate the tumor, just as a marker clip would do.

Factors weakly associated with clip-free localization, such as distribution, are less reproducible, as observed in daily practice. In our study, 43.3% (39/90) of patients had a grade 2 breast tumor with a group of microcalcifications composed strictly of more than 10 units, and our population was comparable to cohorts observed in other studies of breast tumors with microcalcifications treated with neoadjuvant chemotherapy, supporting the external validity of our population [18–20]. This offers the encouraging prospect of avoiding unnecessary marker clips, thereby limiting patient inconvenience and potential complications.

The limitations of our study were, first, its retrospective nature, although this was necessary for an initial approach to this issue. Second, a larger number of readers would have enhanced the internal validity of our study and a larger number of patients included would have enhanced the statistic power.

All these results could suggest the following proposition:

- For grade 2 tumors with more than 10 calcifications: marker clip placement is optional. *We recommend checking the persistence of micro-calcification after diagnostic macrobiopsy.*
- For grade 3 tumors: marker clip placement is recommended, regardless of the initial number of calcifications in the group.

## Conclusion

Our study suggests that more than 10 microcalcifications in a grade 2 breast tumor at the initial diagnosis may obviate the need for a marker clip.

An external validation study by a randomized clinical trial should be performed to confirm our results.

## Data Availability

No datasets were generated or analysed during the current study.

## Abbreviations

AIC: Akaike information criterion
BI-RADS: Breast imaging-reporting and data system
Ca: Calcifications
CI: Confidence interval
ER: Estrogen receptor
HER2: Human epidermal growth factor receptor-2
ICNST: Invasive carcinoma of no special type
ILC: Invasive lobular carcinoma
OR: Odds ratio
PR: Progesterone receptor
SD: Standard deviation
